# Enhanced understanding of cinnamaldehyde’s therapeutic potential in osteoarthritis through bioinformatics and mechanistic validation of its anti-apoptotic effect

**DOI:** 10.3389/fmed.2024.1448937

**Published:** 2024-09-23

**Authors:** Yueyang Sheng, Ruiqing Zhai, Shan Li, Xinyu Wang, Ying Wang, Zhengguo Cui, Chao Wang, Qianqian Wang, Yanzhuo Zhang, Chengai Wu

**Affiliations:** ^1^Department of Molecular Orthopaedics, National Center for Orthopaedics, Beijing Research Institute of Traumatology and Orthopaedics, Beijing Jishuitan Hospital, Capital Medical University, Beijing, China; ^2^College of Bioinformatics Science and Technology, Harbin Medical University, Harbin, China; ^3^Department of Environmental Health, University of Fukui School of Medical Sciences, University of Fukui, Fukui, Japan

**Keywords:** osteoarthritis, cinnamaldehyde, apoptosis, chondrocytes, bioinformatics, gene expression

## Abstract

**Introduction:**

Osteoarthritis (OA) is a globally prevalent joint disorder affecting approximately 240 million individuals worldwide. Cinnamaldehyde, known for its broad anti-inflammatory and anti-aging effects across various cell types, has not been investigated for its potential impact on apoptosis in OA chondrocytes.

**Methods:**

To explore the effectiveness of cinnamaldehyde in mitigating knee osteoarthritis by reducing chondrocyte apoptosis, bioinformatics analysis was first conducted to identify apoptosis-associated differentially expressed genes (APDEGs). Gene expression datasets GSE55235 and GSE114007 were analyzed using weighted gene co-expression network analysis (WGCNA). Gene modules of interest were cross-referenced with APDEGs to identify those specific to OA. LASSO regression analysis was employed to build a risk model, and this model, along with datasets GSE114007, GSE55457, and GSE12021, was validated using ROC analysis. Cellular experiments and blood analyses from OA patients were performed to evaluate the effects of cinnamaldehyde on apoptosis-related gene expression.

**Results:**

Cinnamaldehyde administration was found to rectify the abnormal expression of key apoptosis-related genes in OA patients. Specifically, cinnamaldehyde may affect knee osteoarthritis by regulating apoptosis-related genes such as ZFAND5, BCL6, ELL2, FOSL2, MARCKS, and SGCD. Additionally, three novel apoptotic targets in OA chondrocytes—ZFAND5, ELL2, and SGCD—were identified.

**Discussion:**

These findings provide significant theoretical support for the clinical use of cinnamaldehyde in OA treatment. The discovery of novel apoptotic targets presents new therapeutic possibilities for future OA interventions.

## 1 Introduction

Osteoarthritis (OA) is a progressive joint disorder characterized by the degeneration of cartilage and underlying bone, often resulting in pain, stiffness, and diminished mobility. The etiology of OA is multifaceted, encompassing age (with about 33% of individuals over 75 showing symptomatic and radiographic signs of knee OA), obesity, genetic predisposition, and prior surgical procedures (1). These contributing factors are generally categorized into mechanical, age-related, and genetic influences. Although various treatments such as low-impact aerobic exercises, weight management, acupuncture, NSAIDs, chondroitin sulfate, and surgical interventions are available, they fall short of being ideal pharmacological solutions (2). This underscores the critical need for novel therapeutic strategies to mitigate or reverse the progression of OA.

Natural small molecules, known for their extensive pharmacophore and distinct stereochemical properties, hold significant promise in drug development (3). Cinnamaldehyde, a principal constituent of natural cinnamon oil (4), is a naturally occurring trans-structured acrolein derivative widely utilized across food, spices and medicinal sectors (5). It is recognized for its multiple pharmacological properties, making it a viable candidate in anti-cancer applications. Its potential therapeutic roles have been explored in osteosarcoma (6), oral squamous cell carcinoma, and esophageal squamous cell carcinoma (7). Additionally, cinnamaldehyde is documented for its anti-inflammatory, angiogenesis-promoting, neuroprotective, detoxifying, and antioxidative stress effects (8). Previous research also indicates cinnamaldehyde’s efficacy in treating osteoarthritis and rheumatoid arthritis.

Nonetheless, the complex composition of herbal remedies often limits the scientific understanding of their mechanisms of action, which is essential for pinpointing and enhancing therapeutic effects. Consequently, there is an imperative need to elucidate the mechanisms underlying cinnamaldehyde’s actions to optimize its therapeutic efficacy for disease treatment.

This study leverages bioinformatics techniques to unearth potential targets of cinnamaldehyde in treating knee osteoarthritis (KOA), with a specific emphasis on its ability to inhibit apoptosis. Initial validation was pursued through cellular experiments and clinical blood analysis of OA patients. The insights obtained aim to bolster theoretical support for cinnamaldehyde’s rational clinical use and further experimental exploration.

## 2 Materials and methods

### 2.1 Data retrieval and compilation

Gene expression datasets related to human osteoarthritis were retrieved using the GEOquery package (9) from the NCBI GEO database (10). The data presented in the study are deposited in the GEO repository, accession number GSE55235,^1^ GSE114007,^2^ GSE55457,^3^ GSE12021,^4^ GSE169077,^5^ and GSE1919.^6^ Specifically, GSE55235, GSE55457, and GSE169077 were derived from the GPL96 [HG-U133A] Affymetrix Human Genome U133A Array platform. These datasets comprised 10 osteoarthritis and 10 healthy control samples in GSE55235, RNA-sequenced expression profiles from 10 healthy controls and 10 osteoarthritis patients in GSE55457, and 5 healthy and 6 osteoarthritis samples in GSE169077, respectively. The GSE114007 dataset was obtained from two platforms: the GPL11154 Illumina HiSeq 2000 and the GPL18573 Illumina NextSeq 500, incorporating 18 normal and 20 osteoarthritis samples. GSE12021 leveraged both the GPL96 [HG-U133A] and GPL97 [HG-U133B] Affymetrix platforms, consisting of 10 healthy controls and 10 osteoarthritis cases. Lastly, the GSE1919 dataset was sourced from the GPL91 [HG_U95A] Affymetrix Human Genome U95A Array platform, including 5 normal and 5 osteoarthritis samples. All samples from these datasets were included in this study, with healthy controls categorized as the “Normal” subgroup and osteoarthritis samples classified under the “OA” subgroup. Raw gene expression profiles were acquired using the affy package (11).

### 2.2 Identification of differentially expressed genes and extraction of differential APGs

Differential expression analysis on the GSE55235 dataset was performed utilising GEO2R. Genes that met an adjusted *p*-value threshold of <0.05 and a |FoldChange| of ≥1.5 were flagged as differentially expressed genes (DEGs). Visualization tools such as ggplot2 (12) and ggrepel (13) were employed to create a volcano plot that maps the expression levels of the DEGs in the dataset. Additionally, a heatmap, illustrating the differential expression patterns of these DEGs, was generated using the pheatmap (14) and gplots (15) packages. For the GSE114007 dataset, the LIMMA package (16) facilitated the differential expression analysis, categorizing genes with an adjusted *p*-value <0.05 and a |FoldChange| ≥1.5 as eligible DEGs. Apoptosis-associated gene sets (APGs) were extracted from the GeneCards database (17). These APGs were cross-referenced with the DEGs in the GSE55235 and GSE114007 datasets to identify differentially expressed apoptosis-associated genes (APDEGs).

### 2.3 Functional enrichment and protein–protein interaction network construction

To elucidate the biological functions and signaling pathways associated with APDEGs, Gene Ontology (GO) and Kyoto Encyclopedia of Genes and Genomes (KEGG) pathway enrichment analyses were performed (18, 19). Subsequently, the construction of a protein–protein interaction (PPI) network was undertaken using the STRING database (20), in which the degrees of interaction between nodes were meticulously assessed. Advanced analysis of node correlation properties within this network was conducted employing the Maximal Clique Centrality (MCC) algorithm through the CytoHubba plugin in Cytoscape (21, 22). This allowed for the evaluation of the interconnectivity and reciprocal relationships among the APDEGs, thus providing insights into their potential roles in the biological context.

### 2.4 Weighted gene co-expression network analysis

Weighted gene co-expression network analysis (WGCNA) was utilized to uncover clusters of co-expressed genes correlating with the target phenotype of osteoarthritis (OA). The analysis was executed using the WGCNA package (23). Initially, a scale-free topology of the network was confirmed, followed by the construction of a hierarchical clustering tree. This facilitated the identification of various gene modules. These modules were subsequently correlated with the observed OA phenotypes, further delineating gene clusters that might significantly contribute to the disease’s pathophysiology.

### 2.5 Construction of a risk prognostic model using LASSO regression

The Least Absolute Shrinkage and Selection Operator (LASSO) regression, a method based on linear regression to enhance model prediction accuracy and interpretability, was employed to construct prognostic models. Utilizing the R package glmnet (24), LASSO regression was applied to the GSE55457 dataset with the goal of identifying significant influencing factors and establishing a robust risk prognostic model.

### 2.6 Validation of the risk prognostic model

The prognostic models developed for OA risk using LASSO regression were subjected to validation (25). This was accomplished using receiver operating characteristic (ROC) curves. The datasets GSE114007, GSE55457, and GSE12021 served as the basis for validation. Risk classification thresholds were set according to the median value, and ROC values were computed employing the R package ROCR (26), allowing for precise assessment of model performance.

### 2.7 Study population

This study incorporated a cohort comprising 5 patients aged between 55 and 75 years diagnosed with primary osteoarthritis, categorized under the OA group. The control group consisted of 9 healthy volunteers ranging from 24 to 60 years old, with no history of OA or associated clinical symptoms. Ethical approval for this research was granted by the ethics committee of Beijing Jishuitan Hospital (ethics code: 201611-03), ensuring compliance with the Declaration of Helsinki.

### 2.8 Sample handling

Plasma samples were collected from nine healthy individuals and five patients with knee osteoarthritis (OA). Blood samples were drawn using venipuncture into EDTA tubes to inhibit clotting. Subsequently, these samples were centrifuged at 1,500 × g for 15 min at 4°C to facilitate plasma separation. The isolated plasma was then aliquoted with care and stored at −80°C until further analysis to preserve sample integrity. For RNA extraction, total RNA was isolated from the plasma employing TRIzol LS Reagent, adhering strictly to the manufacturer’s instructions. The protocol included the addition of TRIzol LS to the plasma, vigorous mixing, and subsequent incubation for phase separation. Following centrifugation, the aqueous phase, containing the RNA, was transferred to a new tube. RNA was precipitated using isopropanol, washed with 75% ethanol, air-dried, and finally dissolved in RNase-free water. The RNA quality was meticulously assessed to ensure adherence to quality control standards, including an A260/A280 ratio of approximately 1.8–2.0, indicative of high RNA purity, and an adequate concentration level. Only RNA samples meeting these stringent quality criteria were used in the subsequent gene expression analyses, thereby ensuring the reliability of our data.

### 2.9 Sources of normal and OA chondrocytes

Articular cartilage samples were procured from the lateral tibial plateau within 1 h of surgery. These tissues were promptly processed to isolate the chondrocytes, which were then utilized in subsequent experiments.

### 2.10 Reagents and cell culture

CCCP (CAS:555-60-2) was sourced from Solarbio, China. The cells were maintained at 37°C and 5% CO_2_ in a complete cell culture medium consisting of Dulbecco’s Modified Eagle’s Medium (DMEM)/F-12, supplemented with fetal bovine serum and antibiotics in a 100:10:1 ratio. Upon reaching 80–90% confluence, cells were passaged biweekly at a 1:3 ratio. Cells from passages three to five were selected for the ensuing experiments.

### 2.11 Quantitative real-time PCR analysis

Total RNA was extracted from chondrocytes and used to synthesize cDNA. Real-time PCR was conducted using the Applied Biosystems 7500 Real Time PCR System, following the manufacturer’s protocol. Gene expression in blood samples was quantified employing the SYBR Green method on an ABI 7900HT fluorescent quantitative PCR instrument (ABI, America). GAPDH was utilized as the internal control. Relative gene expression levels were calculated using the comparison Ct (2^−ΔΔCT^) method. Specific primer sequences for the genes studied are listed in Table 1.

**Table 1:** Sequences of primers used in qRT-PCR.

Genes		Sequences
MARCKS	Forward	AGCCCGGTAGAGAAGGAGG
	Reverse	TTGGGCGAAGAAGTCGAGGA
ZFAND5	Forward	GTGGACTTCACCGTTACTCTG
	Reverse	TTTCAGCCACAACAACTGGAT
BCL6-F	Forward	GGAGTCGAGACATCTTGACTGA
	Reverse	ATGAGGACCGTTTTATGGGCT
FOSL2-F	Forward	CAGAAATTCCGGGTAGATATGCC
	Reverse	GGTATGGGTTGGACATGGAGG
ELL2-F	Forward	CATCACCGTACTGCATGTGAA
	Reverse	ACTGGATTGAAGGTCGAAAAGG
COL2A1	Forward	GAAGGATGGCTGCACGAAAC
	Reverse	CGGGAGGTCTTCTGTGATCG
SGCD	Forward	GCGGAAACGATGCCTGTATTT
	Reverse	TGGCGTAGAGAGGTTGTAAGAAG
IL-6	Forward	ACTCACCTCTTCAGAACGAATTG
	Reverse	CCATCTTTGGAAGGTTCAGGTTG
BAX	Forward	CCCGAGAGGTCTTTTTCCGAG
	Reverse	CCAGCCCATGATGGTTCTGAT
BCL-2	Forward	CCCGAGAGGTCTTTTTCCGAG
	Reverse	CCAGCCCATGATGGTTCTGAT
BCL-2	Forward	GGTGGGGTCATGTGTGTGG
	Reverse	CGGTTCAGGTACTCAGTCATCC
GAPDH	Forward	AAGGGTCATCATCTCTGCCC
	Reverse	GTGAGTGCATGGACTGTGGT

### 2.12 TUNEL assay

To assess chondrocyte apoptosis, the TUNEL method was applied. Cells were fixed in 4% paraformaldehyde for 15–20 min and washed thrice with PBS. Chondrocytes were permeabilized with 0.1% Triton X-100 at 4°C for 10 min and stained with DAPI. Apoptotic rates were determined by quantifying the percentage of TUNEL-positive cells under a fluorescence microscope.

### 2.13 ROS detection assay

Chondrocytes were plated at a density of 15,000 cells per well and co-cultured with 50 μM CCCP for 24 h. After washing the cells 2–3 times, they were incubated with 10 μM DCFH-DA (Beyotime, China) at 37°C for at least 20 min in the dark. ROS generation was observed under a fluorescence microscope.

### 2.14 Apoptosis detection by flow cytometry

Human primary chondrocytes were cultured in 6-well plates at a density of 1.6 × 10^5^ cells/well. After 12 h of incubation, cells were treated with cinnamaldehyde and 10 ng/mL of IL-1β (Sigma-Aldrich, United States) for 24 h. Apoptosis was evaluated using a PE Annexin V Apoptosis Kit (BD, 559763). Cells (1 × 10^6^/mL) were resuspended in 100 μL of binding buffer from the kit, with 5 μL of Annexin V-FITC and 5 μL of propidium iodide (PI) subsequently added. Following a 20 min incubation at room temperature in the dark, cells were analyzed promptly using a BD CantoII flow cytometer. Data acquisition and analysis were conducted with FlowJo software. Detection channels included FITC for Annexin V-FITC and PE for PI, enabling quantification of live, early apoptotic, late apoptotic/necrotic, and necrotic cells. Apoptotic percentages were computed and compared across different experimental conditions.

### 2.15 Western blot analysis

Total protein was extracted from cells using RIPA lysis buffer (Keygen Bio Tech, China), adhering strictly to the manufacturer’s protocol. Protein concentrations were quantified employing the BCA Protein Assay Kit (Beyotime, China). Proteins were then separated by SDS-PAGE and electrotransferred onto polyvinylidene fluoride (PVDF) membranes. The membranes were initially blocked with 5% skim milk for 2 h, followed by overnight incubation at 4°C with primary antibodies targeting BCL6 (Proteintech-21187-1-AP), SGCD (ab137101), MARCKS (Proteintech-20661-1-AP), FOSL2 (CST-59971), ELL2 (Proteintech-12727-1-AP), ZFAND5 (Bioss-bs-10142R), BCL-2 (ab238042), and *β*-actin (ab8226). Afterward, the membranes were rinsed three times with tris-buffered saline containing 0.1% Tween^®^ 20, with each wash lasting 6 min. The membranes were then incubated with horseradish peroxidase-conjugated secondary antibodies—either mouse IgG (Biorigin-BN20602) or rabbit IgG (Biodee-DE0601)—for 1 h at room temperature. Immunoreactive bands were visualized using an enhanced chemiluminescence (ECL) substrate (Sage Creation) and quantified with ImageJ software.

### 2.16 Immunofluorescence staining

Following various treatments, cells were fixed with 4% paraformaldehyde (PFA) for 15 min at room temperature, followed by permeabilization with 0.2% Triton X-100 in PBS for 20 min. To reduce nonspecific binding, cells were blocked with 1% bovine serum albumin (BSA) in PBS for 1 h. Cells were then incubated with primary antibodies against BCL6 (Proteintech-21187-1-AP), SGCD (ab137101), MARCKS (Proteintech-20661-1-AP), FOSL2 (CST-59971), ELL2 (Proteintech-12727-1-AP) and ZFAND5 (Bioss-bs-10142R) for 2 h. After washing with PBS, cells were incubated with Alexa Fluor 488-conjugated anti-rabbit IgG secondary antibodies (Abcam-ab150077) for 1 h. Nuclei were stained with DAPI, and fluorescence images were captured using a high-content screening system (Molecular Imaging Devices, United States).

### 2.17 Drug mechanism study

To elucidate the interaction between key genes associated with OA and cinnamaldehyde, external datasets and databases were employed for exploratory analysis (27). This facilitated the construction of a mechanistic model predicting the efficacy of cinnamaldehyde in treating OA. Molecular docking was performed using Autodock Vina 1.2.2, a state-of-the-art *in silico* protein–ligand docking software (28). The molecular structure of cinnamaldehyde was retrieved from the PubChem Compound database^7^ (29). The 3D coordinates of target proteins were downloaded from the Protein Data Bank (PDB).^8^ Prior to docking, all protein and ligand files were converted into PDBQT format, with water molecules excluded and polar hydrogen atoms added. The docking grid box was precisely centered to encompass the active domain of each protein and to ensure free molecular movement within dimensions of 30 Å × 30 Å × 30 Å, with a grid point distance set at 0.05 nm. Docking simulations were conducted using AutoDock Vina 1.2.2.^9^

### 2.18 Surface plasmon resonance analysis

The binding interactions between cinnamaldehyde and the protein BCL6 were investigated using a Biacore 8 K biosensor system and surface plasmon resonance (SPR) spectroscopy. The target protein was immobilized on a CM5 sensor chip through amine-coupling. Binding assays were carried out at a constant temperature of 25°C and a flow rate of 30 μL/min. Solutions of the compound were prepared at concentrations ranging from 2 to 10 mM and introduced in a running buffer across the immobilized protein at corresponding gradient concentrations. The kinetic dissociation constants (KD) for each interaction were computed using Biacore’s Insight Evaluation Software.

### 2.19 Statistical analysis

Statistical analyses were performed using GraphPad Prism version 9.0 and R software version 4.0.0. Differences among multiple groups were assessed using one-way Analysis of Variance (ANOVA). Comparisons between two groups were made using student’s *t*-test. A *p*-value of less than 0.05 was considered to denote statistical significance.

## 3 Results

### 3.1 Analysis of variance and screening of APDEGs

**Figure 1:**
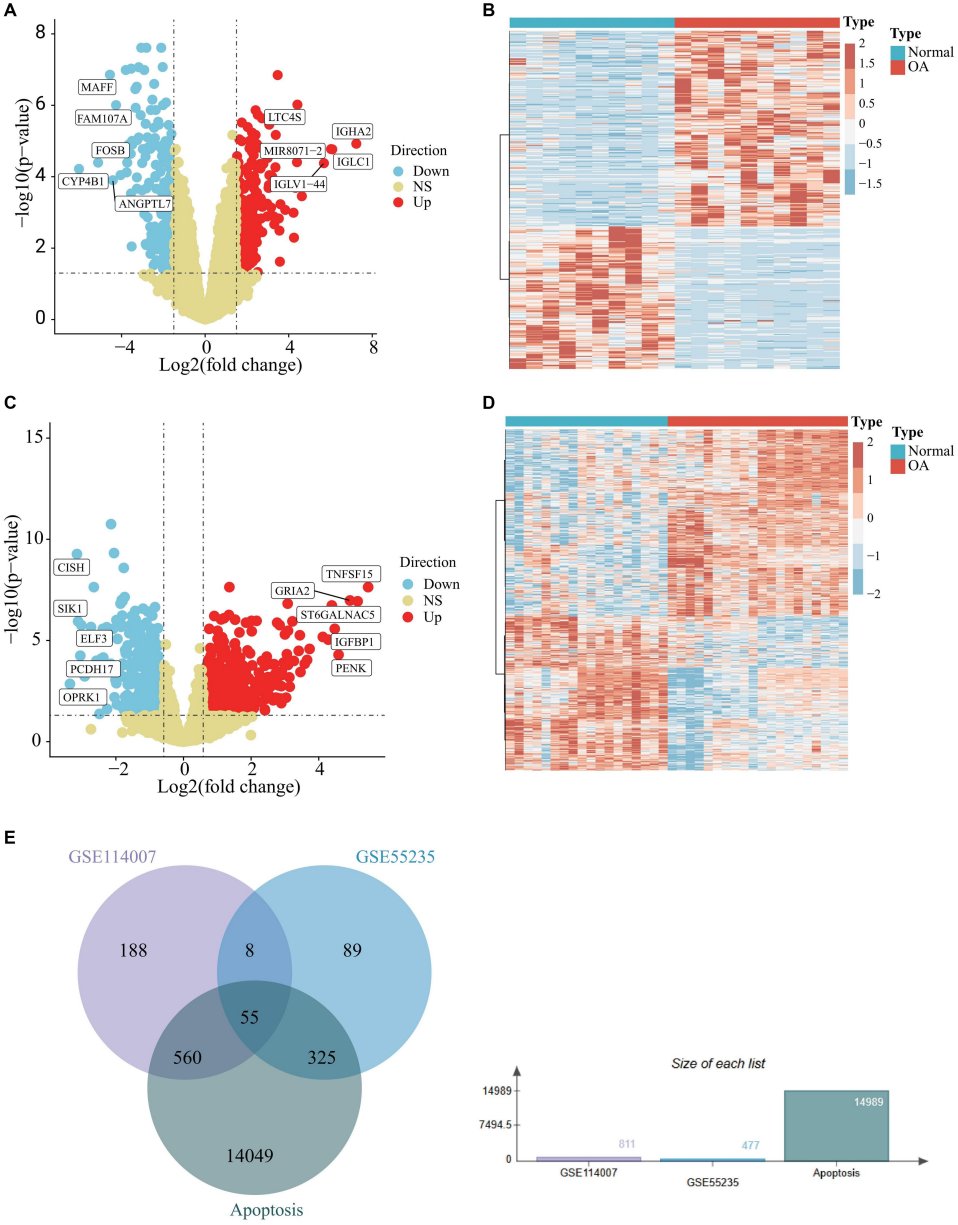
Differential analysis of GSE55235 and GSE114007 and identification of APDEGs. **(A)** A volcano plot of GSE55235, with up-regulated differential genes shown in red, down-regulated differential genes shown in blue, and no differential genes shown in yellow. **(B)** A heatmap of DEGs of GSE55235, where blue represents the “Normal” group, red represents the “OA” group. **(C)** A volcano plot of GSE114007, with up-regulated differential genes shown in red, down-regulated differential genes shown in blue, and non-differential genes shown in yellow. **(D)** A heatmap of DEGs in GSE114007, where blue represents the “Normal” group, red represents the “OA” group. **(E)** A Venn diagram showing the intersection of DEGs in GSE55235, DEGs in GSE114007, and apoptosis-related genes.

To analyze osteoarthritis data, we selected datasets GSE55235, GSE114007, GSE55457, and GSE12021 from the GEO data platform. Each dataset’s expression matrices were standardized individually. Differential analysis of GSE55235, performed using GEO2R, identified 477 differential genes meeting the criteria of an adjusted *p*-value <0.05 and a |fold change | ≥1.5, comprising 276 up-regulated and 201 down-regulated genes relative to normal samples. These findings are depicted graphically in a volcano plot (Figure 1A) and a heat map (Figure 1B). Differential analysis of GSE114007, conducted using the LIMMA package, revealed 811 differential genes, with 445 up-regulated and 366 down-regulated. These results are also visualized in a volcano plot (Figure 1C) and heat map (Figure 1D). Cross-referencing DEGs from GSE55235 and GSE114007 with apoptosis-related genes (APDEGs) from GeneCards resulted in 55 APDEGs exhibiting significant expression differences (Figure 1E), highlighting specific APDEGs’relevance in osteoarthritis and laying a foundation for further functional analysis.

### 3.2 Enrichment analysis of PRGs and PPI network construction

**Figure 2:**
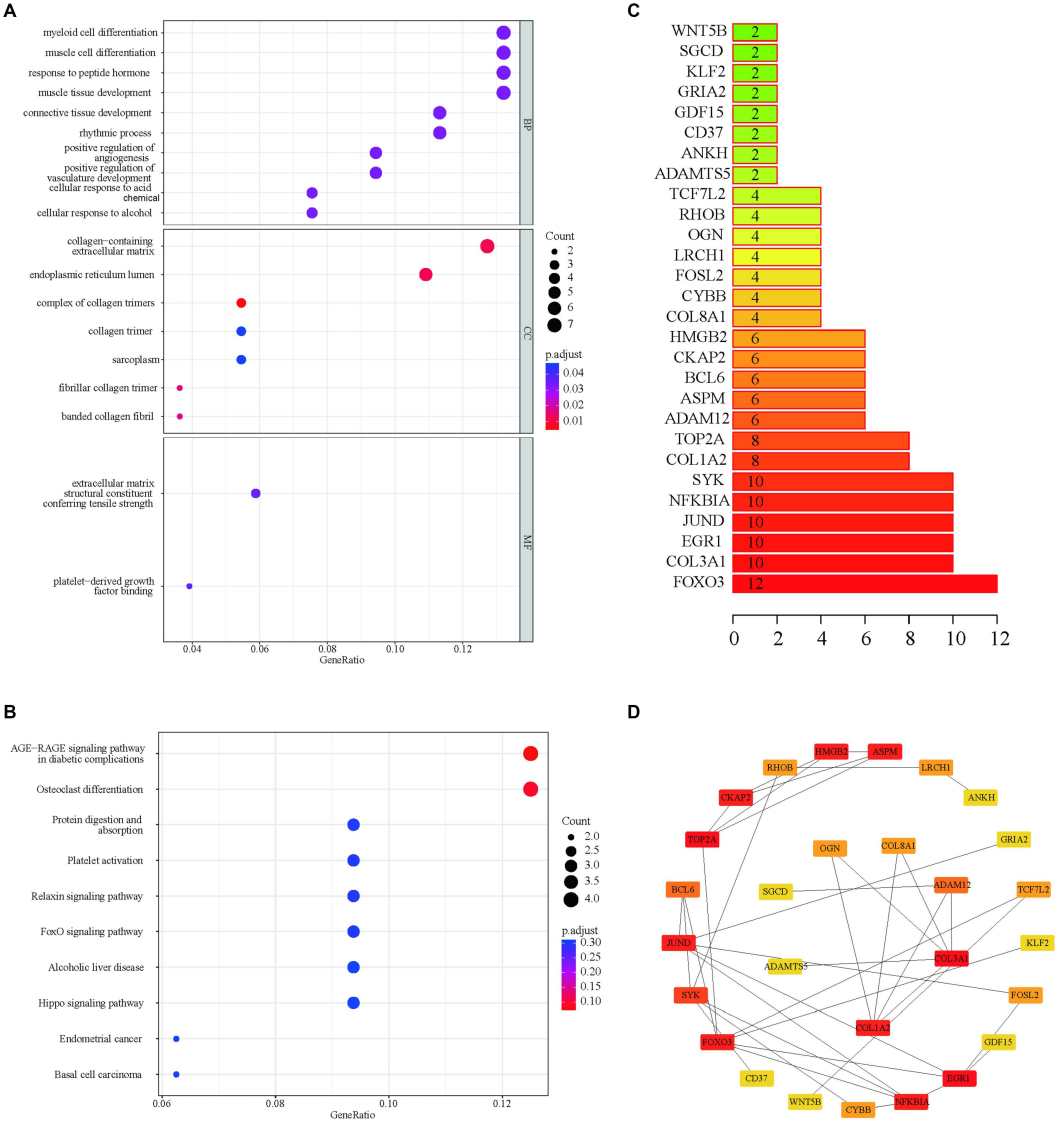
GO enrichment, KEGG enrichment and PPI network construction based on APDEGs. **(A)** The GO biological function enrichment analysis of APDEGs. The *X*-axis represents the proportion of gene numbers under this item, and the adjusted *p*-value of the enrichment result is expressed through color: the darker the color, the smaller the adjusted *p*-value. The size of the dots represents the number of enriched genes. **(B)** KEGG enrichment pathway analysis. The size of the dots indicates the number of enriched genes, and the color of the dots indicates the adjusted *p*-value: the redder the color, the smaller the adjusted *p*-value; the purer the color, the larger the adjusted *p*-value. **(C)** Statistics of the number of protein interactions in the APDEGs. **(D)** The hub node network map of APDEGs using the MCC algorithm.

Functional enrichment analyses of the 55 APDEGs utilized the Gene Ontology (GO) and Kyoto Encyclopedia of Genes and Genomes (KEGG) databases (Figures 2A,B). This analysis revealed APDEGs’ involvement in biological processes such as myeloid cell differentiation, muscle cell differentiation, and the formation of collagen-containing extracellular matrix (Figure 2A). Additionally, KEGG pathways implicated included AGE-RAGE signaling, osteoclast differentiation, and protein digestion and absorption (Figure 2B). Interaction among the APDEGs was visualized through network analysis using the STRING database; hub genes were identified using the MCC algorithm (Figures 2C,D), providing crucial insights into biological roles and pathways involving APDEGs in osteoarthritis, thereby aiding the development of targeted therapeutic strategies.

### 3.3 Construction of the WGCNA network and identification of OA modules

**Figure 3:**
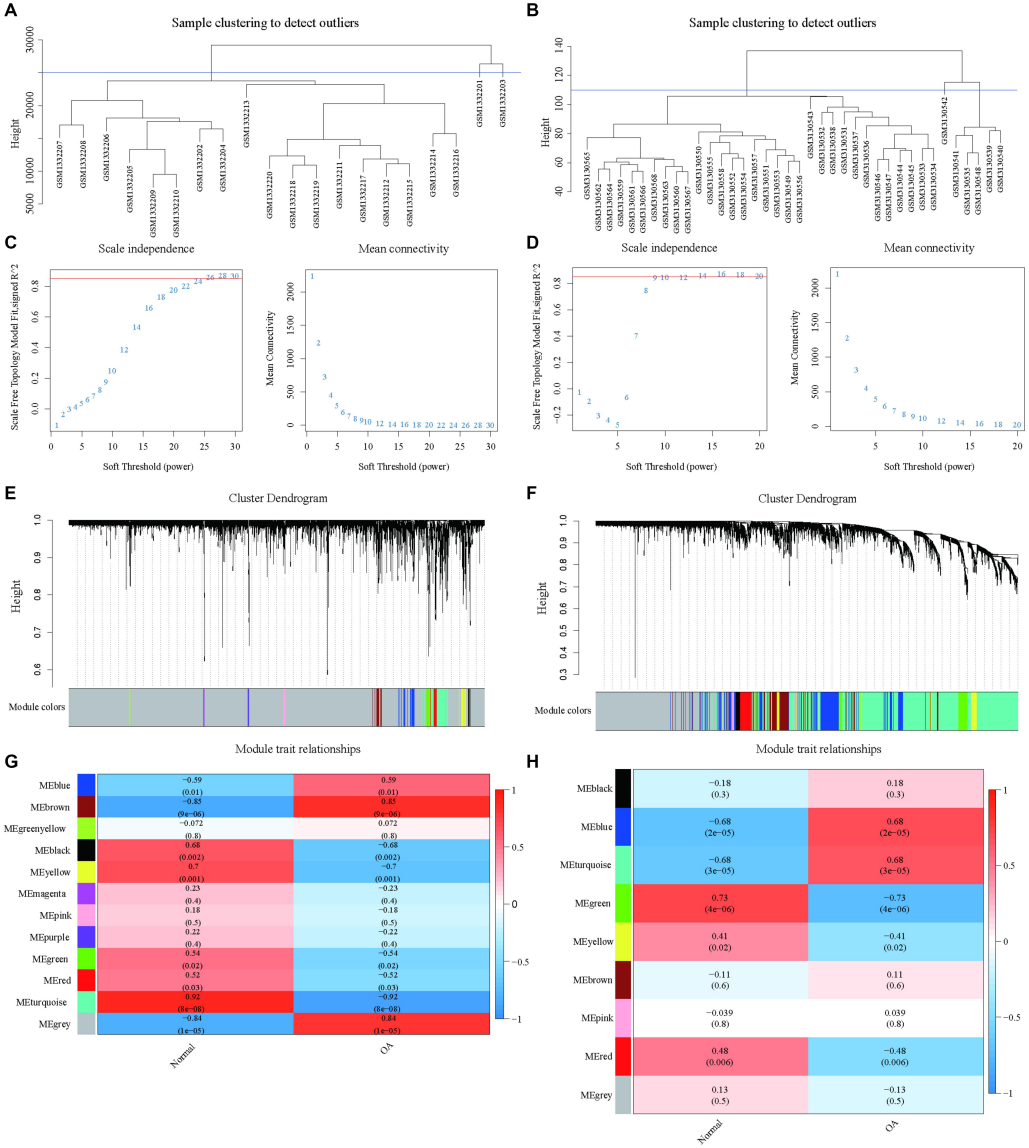
Construction of WGCNA network based on datasets GSE55235 and GSE114007 with identification of OA modules. **(A)** The clustering dendrogram of 20 samples in the GSE55235 data. **(B)** The clustering dendrogram of 38 samples in the GSE114007 data. **(C)** The identification of soft thresholds for WGCNA (weighted correlation network analysis) in the GSE55235 data. The left panel shows the scale-free metric analysis for various soft threshold powers (β), and the right panel shows the average connectivity analysis for various soft threshold powers. **(D)** The determination of soft thresholds for WGCNA in the GSE114007 data. **(E)** The tree diagram (1-TOM) of all genes clustered based on dissimilarity measurements for GSE55235. The colored bands show the results obtained from the automated single block analysis. **(F)** The tree diagram (1-TOM) of all genes clustered based on dissimilarity measurements for GSE114007. The color band shows the results obtained from the automated single block analysis. **(G)** A heatmap of the correlation between GSE55235 module feature genes and clinical features and a heatmap of correlation between GSE114007 module feature genes and clinical features.

Weighted gene co-expression network analysis (WGCNA) was applied to the gene expression matrix derived from GSE55235, encompassing data from 10 OA and 10 healthy control samples. Initial Pearson correlation analysis identified and excluded two outlier samples (Figure 3A). A soft threshold of *β* = 26, demonstrating optimal scale-free, topology “R^2^,” was chosen for network construction (Figure 3C). This analysis identified 12 modules, with specific modules (blue, brown, black, yellow, green, red, turquoise, and grey) significantly associated with pathological grading and selected for further clinical analysis (Figures 3E,G).

A similar approach with dataset GSE114007 identified nine significant modules after exclusion based on clustering analysis (Figures 3B,D,F,H), underscoring key gene modules linked with osteoarthritis pathology to be explored for clinical relevance.

### 3.4 Risk model construction based on APDEGs

**Figure 4:**
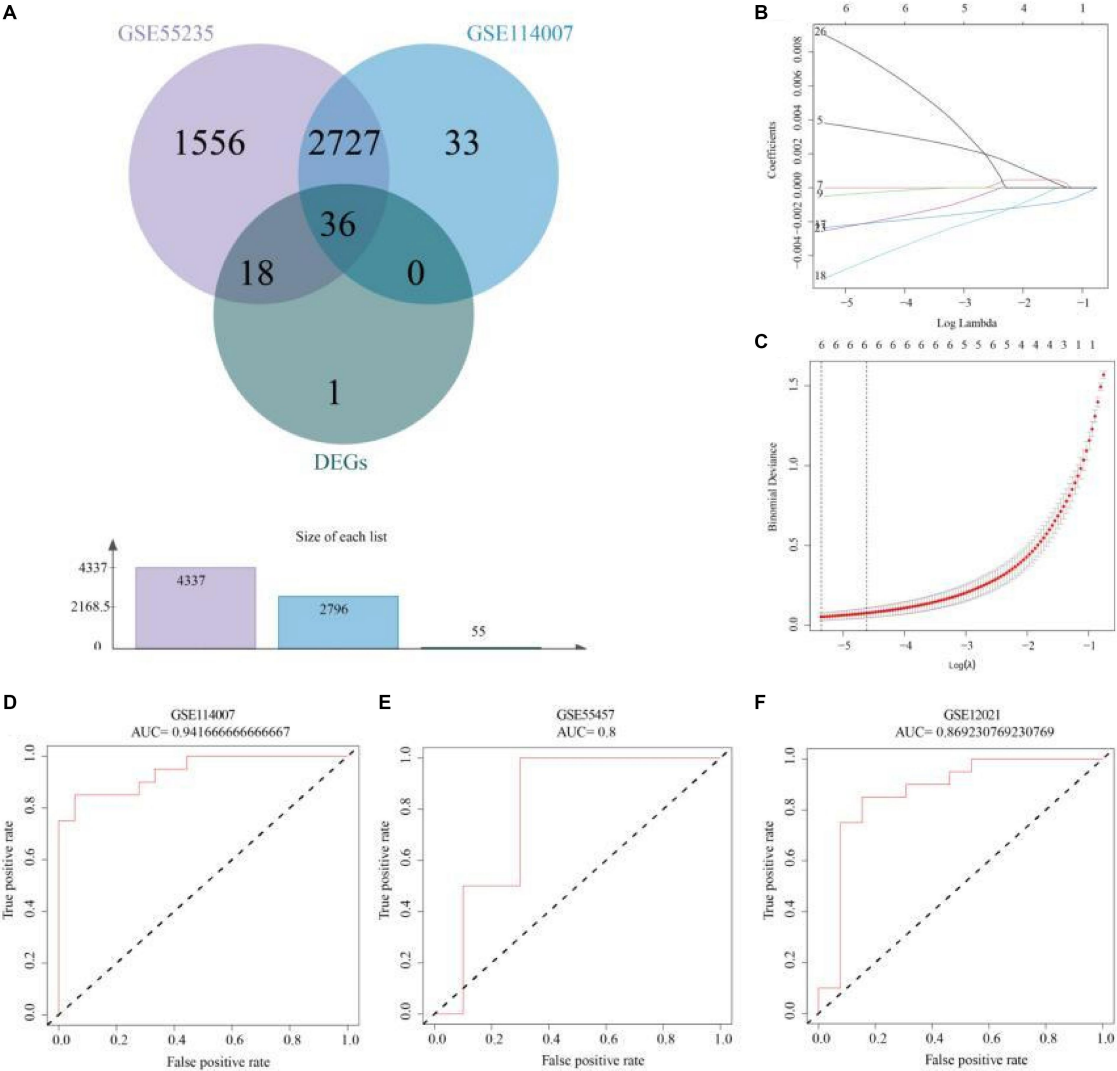
Risk model construction and validation based on APDEGs. **(A)** A Venn diagram showing the intersection genes of GSE55235 and GSE114007 in WGCNA yielding modular genes significantly associated with OA and APDEGs. **(B)** Coefficient plots, displaying the gene coefficients with log lambda values in the horizontal axis and gene coefficients in the vertical axis. **(C)** Feature selection using the LASSO regression model by 10-fold cross-validation and minimum criterion to generate coefficient profiles based on log (lambda) sequences. **(D)** The ROC curve indicates that the accuracy of risk model prediction of GSE114007 is 0.94. **(E)** The ROC curve indicates that the accuracy of the risk model’s prediction for GSE55457 is 0.80. **(F)** The ROC curve indicates that the accuracy of risk model prediction for GSE12021 is 0.87.

APDEGs significantly associated with OA from datasets GSE55235 and GSE114007 were further examined, leading to the identification of 55 APDEGs. EVenn was then employed to discern 36 APDEGs linked specifically to OA (Figure 4A). These genes underwent feature selection through 10-fold cross-validation using the LASSO regression model based on the GSE55235 dataset (Figures 4B,C). Significant model genes included *MARCKS*, *ZFAND5*, *BCL6*, *FOSL2*, *ELL2*, and *SGCD*. Validation of the risk prognosis model via ROC curve analysis from datasets GSE114007, GSE55457, and GSE12021 showed AUCs over 0.8 (Figures 4D–F), evidencing robust model efficacy in forecasting osteoarthritis prognosis, and highlighting its potential utility in clinical settings.

### 3.5 Cinnamaldehyde’s effect on the viability of human OA chondrocytes

**Figure 5:**
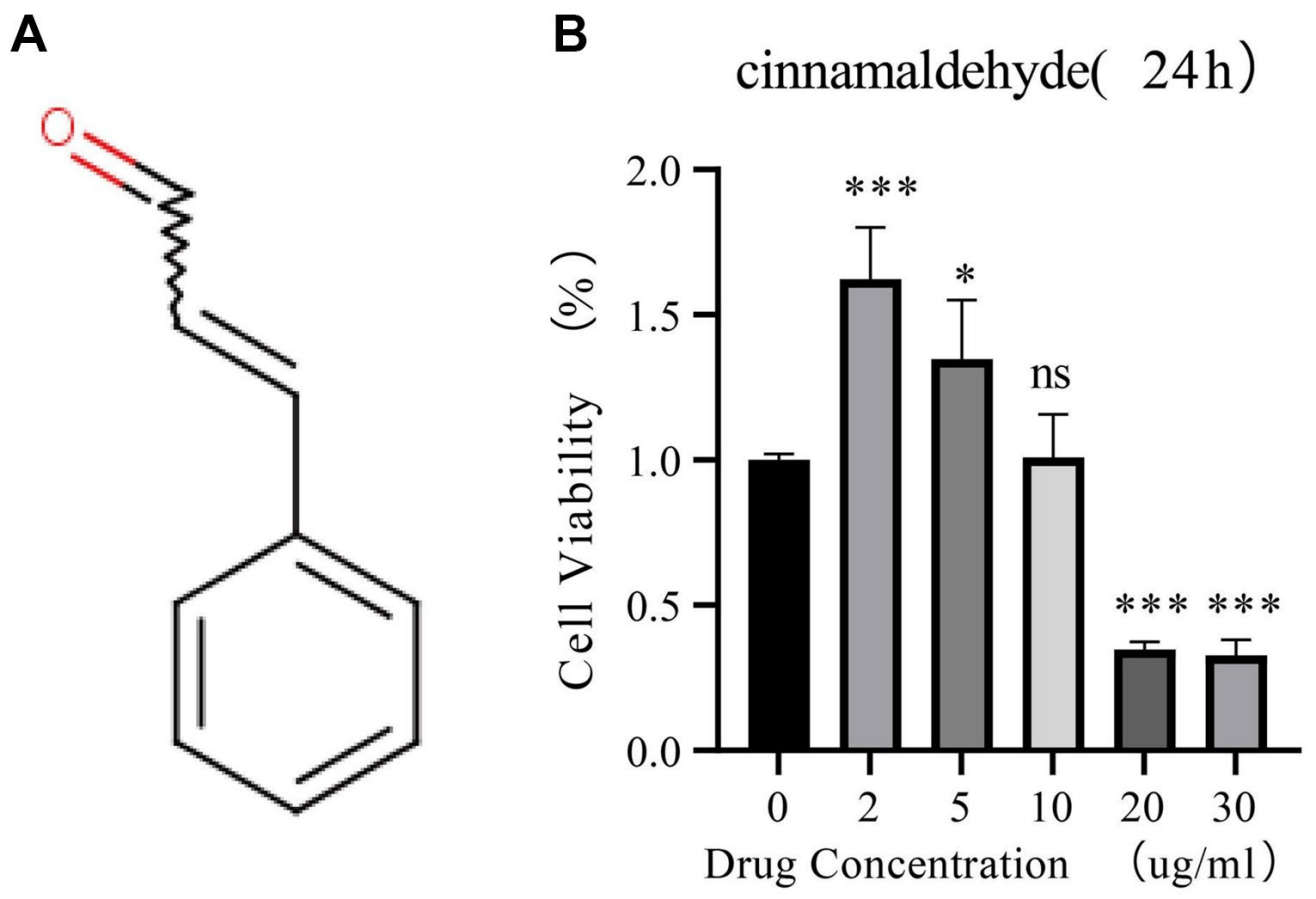
The effects of cinnamaldehyde on cell viability of OA chondrocytes. **(A)** The chemical structure of cinnamaldehyde. **(B)** OA chondrocytes were treated with cinnamaldehyde (0, 2, 5, 10, 20 or 30 μg/mL) for 24 h, followed by assessment of cell viability by CCK-8 assay. ^*^p < 0.05, ^**^p < 0.01, ^***^p < 0.001, and ^****^p < 0.0001. All experiments were repeated at least three times.

This study investigated the impact of cinnamaldehyde on the viability of human chondrocytes derived from patients with OA. The chemical structure of cinnamaldehyde is depicted in Figure 5A. Initially, chondrocytes were treated with various concentrations of cinnamaldehyde (2, 5, 10, 20, and 30 μg/mL) for 24 h. Utilizing the Cell Counting Kit-8 (CCK-8) assay, our results indicated that exposure to 2 and 5 μg/mL significantly enhanced the viability of OA chondrocytes compared to control treatments. In contrast, a concentration of 10 μg/mL did not significantly impact cell viability. Notably, concentrations of 20 and 30 μg/mL produced a concentration-dependent inhibition of OA chondrocytes’ viability (Figure 5B). These findings suggest that cinnamaldehyde exerts a biphasic effect on the viability of human OA chondrocytes, enhancing cell viability at lower concentrations (2–5 μg/mL) and inhibiting it at higher concentrations (20–30 μg/mL). Therefore, the therapeutic potential of cinnamaldehyde for OA likely depends on its concentration, warranting further research to elucidate its mechanisms of action and to establish optimal dosages for therapeutic applications.

### 3.6 Suppression of inflammatory and apoptosis-related gene expression by cinnamaldehyde in OA chondrocytes

**Figure 6:**
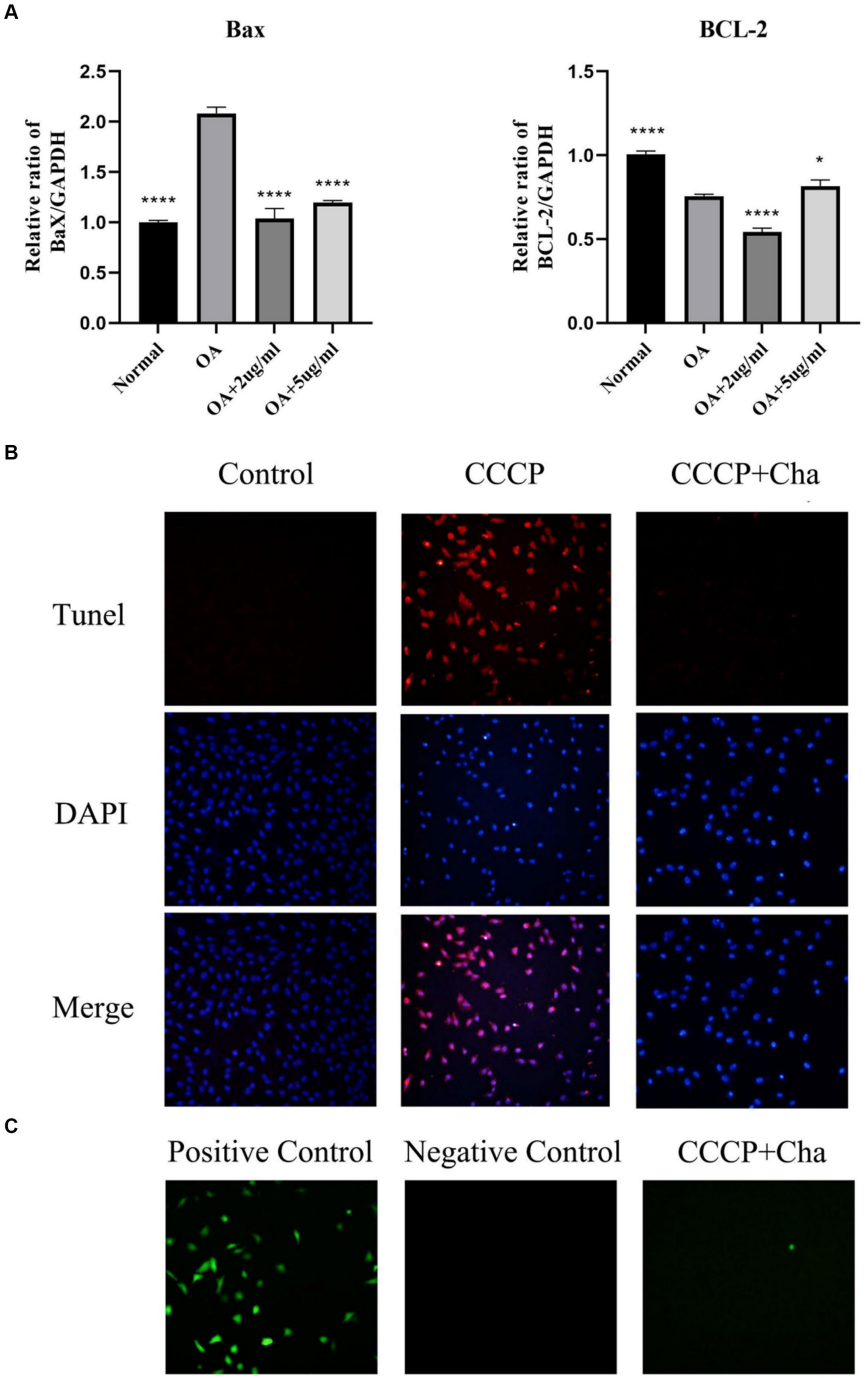
Cinnamaldehyde alleviated apoptosis induced by CCCP. **(A)** The mRNA expressions of Bax and Bcl-2 in OA chondrocytes were quantified using qRT-PCR. **(B)** The TUNEL staining of chondrocytes with CCCP (50 μM) and cinnamaldehyde (5 μg/mL) for 24 h and the corresponding proportion of apoptotic cells. **(C)** ROS detection of chondrocytes with CCCP (50 μM) and cinnamaldehyde (5 μg/mL) for 24 h. ^*^p < 0.05, ^**^p < 0.01, ^***^p < 0.001, and ^****^p < 0.0001. All experiments were repeated at least three times.

This part of the study focused on the inhibitory effects of cinnamaldehyde on the expression of inflammatory and APDEGs in OA chondrocytes. To evaluate the anti-apoptotic potential of cinnamaldehyde, assays including real-time PCR (qRT-PCR) (Figure 6A), TUNEL (Figure 6B), and ROS assay (Figure 6C) were conducted. Notably, treatment with carbonyl cyanide 3-chlorophenylhydrazone (CCCP) increased ROS levels and apoptosis in chondrocytes, which was attenuated by cinnamaldehyde treatment, suggesting its protective effects. Flow cytometry results further supported this observation, showing that cinnamaldehyde can significantly reduce apoptosis in osteoarthritic chondrocytes (Supplementary Figure S1A). Additionally, qRT-PCR analysis indicated a dose-dependent decrease in the expression of the pro-apoptotic gene *Bax* and an increase in the anti-apoptotic gene *Bcl-2* following cinnamaldehyde treatment. When compared to normal chondrocytes, IL-1β-stimulated chondrocytes exhibited an increased expression of Bcl-2, which was effectively reversed by cinnamaldehyde, aligning with changes in RNA expression (Supplementary Figure S1B). The dynamic interaction between Bax, which promotes apoptosis, and Bcl-2, which extends chondrocyte lifespan, underscores cinnamaldehyde’s capacity to mitigate apoptosis in OA chondrocytes.

### 3.7 Differential expression of key genes in OA and normal plasma and chondrocytes

**Figure 7:**
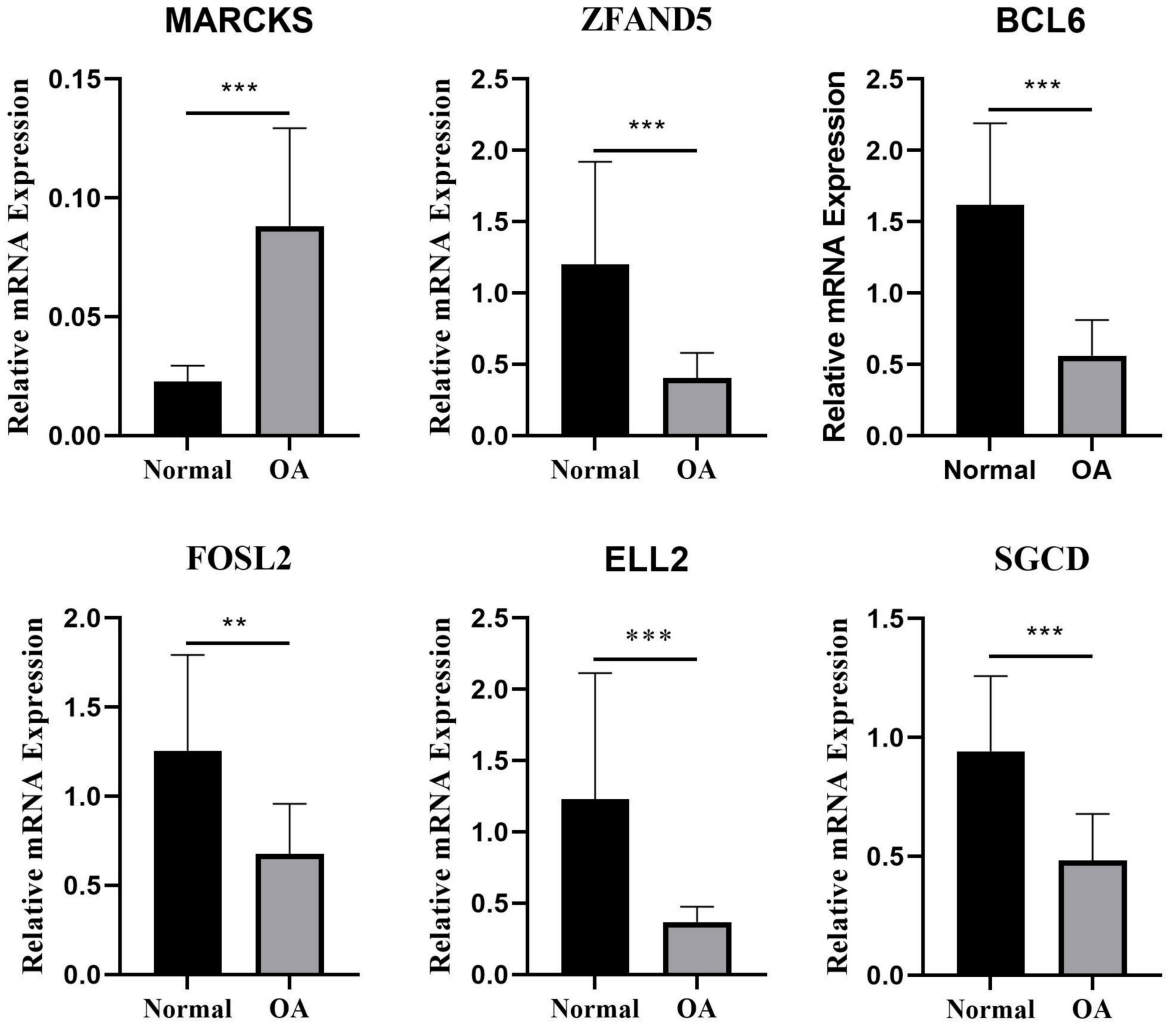
The relative mRNA expression of select DEGs in plasma. The relative mRNA expression of DEGs between osteoarthritis and normal chondrocytes in plasma ^*^p < 0.05, ^**^p < 0.01, ^***^p < 0.001, and ^****^p < 0.0001.

**Figure 8:**
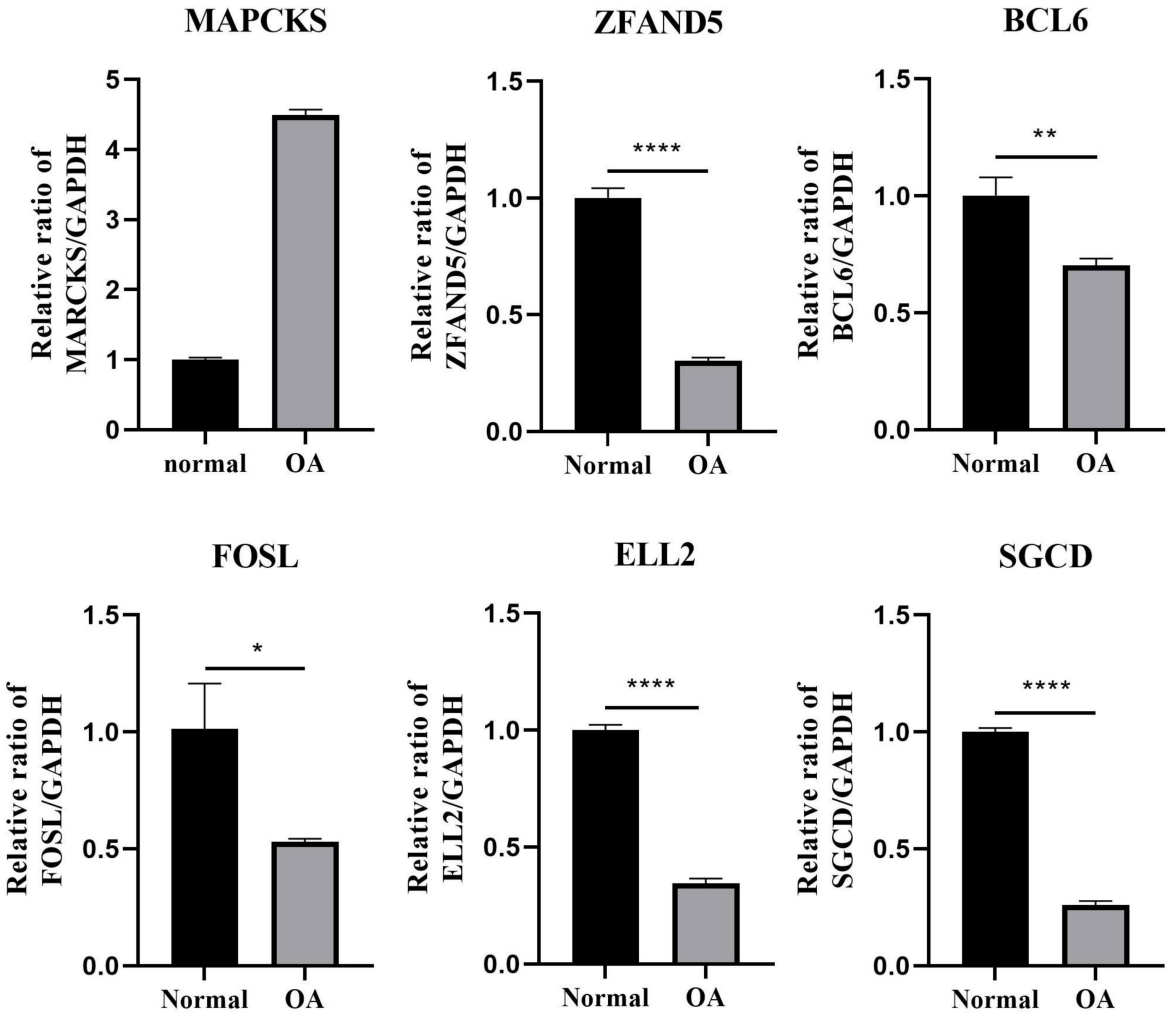
The relative mRNA expression of select DEGs in OA cell. The relative mRNA expression of DEGs between osteoarthritis and normal chondrocytes in OA cell ^*^p < 0.05, ^**^p < 0.01, ^***^p < 0.001, and ^****^p < 0.0001. All experiments were repeated at least three times.

In this study, plasma samples from 9 healthy volunteers and 5 patients with OA were analyzed to assess the expression levels of key DEGs. Notably, *MARCKS* was founded to be significantly upregulated in the OA patients compared to the controls (*p* = 0.0002; Figures 7, 8). Conversely, *ZFAND5*, *BCL6*, *FOSL2*, and *ELL2* were downregulated in OA patients compared to healthy controls (Figures 7, 8), consistent with previous bioinformatics analyses.

### 3.8 Regulation of apoptosis-related genes in OA chondrocytes by cinnamaldehyde

**Figure 9:**
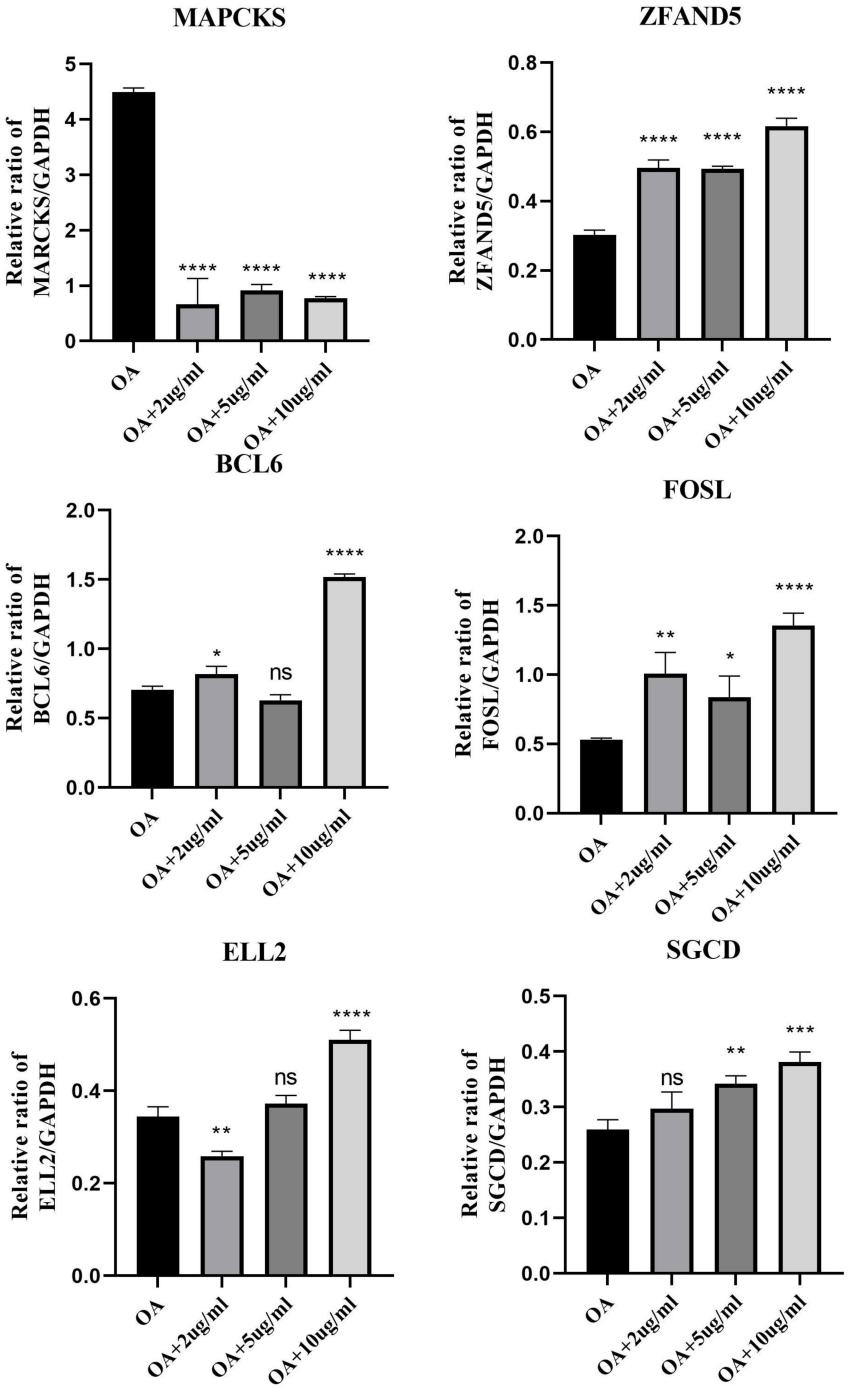
The mRNA expression of select DEGs with cinnamaldehyde treatment. The mRNA expressions of DEGs in OA chondrocytes were quantified using qRT-PCR after they were exposed to 2, 5 or 10 μg/mL of cinnamaldehyde for 24 h. ^*^p < 0.05, ^**^p < 0.01, ^***^p < 0.001, and ^****^p < 0.0001. All experiments were repeated at least three times.

This segment of the study explored the regulatory effects of cinnamaldehyde on apoptosis-related genes in OA chondrocytes. OA chondrocytes were treated with cinnamaldehyde for 24 h, with untreated cells serving as controls. Post-treatment, RNA extraction and subsequent analyses showed that cinnamaldehyde significantly inhibited the expression of *MARCKS* and increased the expression of *ZFAND5*, *BCL6*, *FOSL2*, *ELL2*, and *SGCD* (Figure 9). Protein expressions detected through immunofluorescence staining (Supplementary Figure S2) and Western blot analysis (Supplementary Figure S3) corroborated the PCR results, reinforcing cinnamaldehyde’s regulatory impact on these genes. Notably, the effects of cinnamaldehyde on *ZFAND5*, *ELL2*, and *SGCD* were significantly dose-dependent.

### 3.9 Correlation verification and drug mechanism prediction

**Figure 10:**
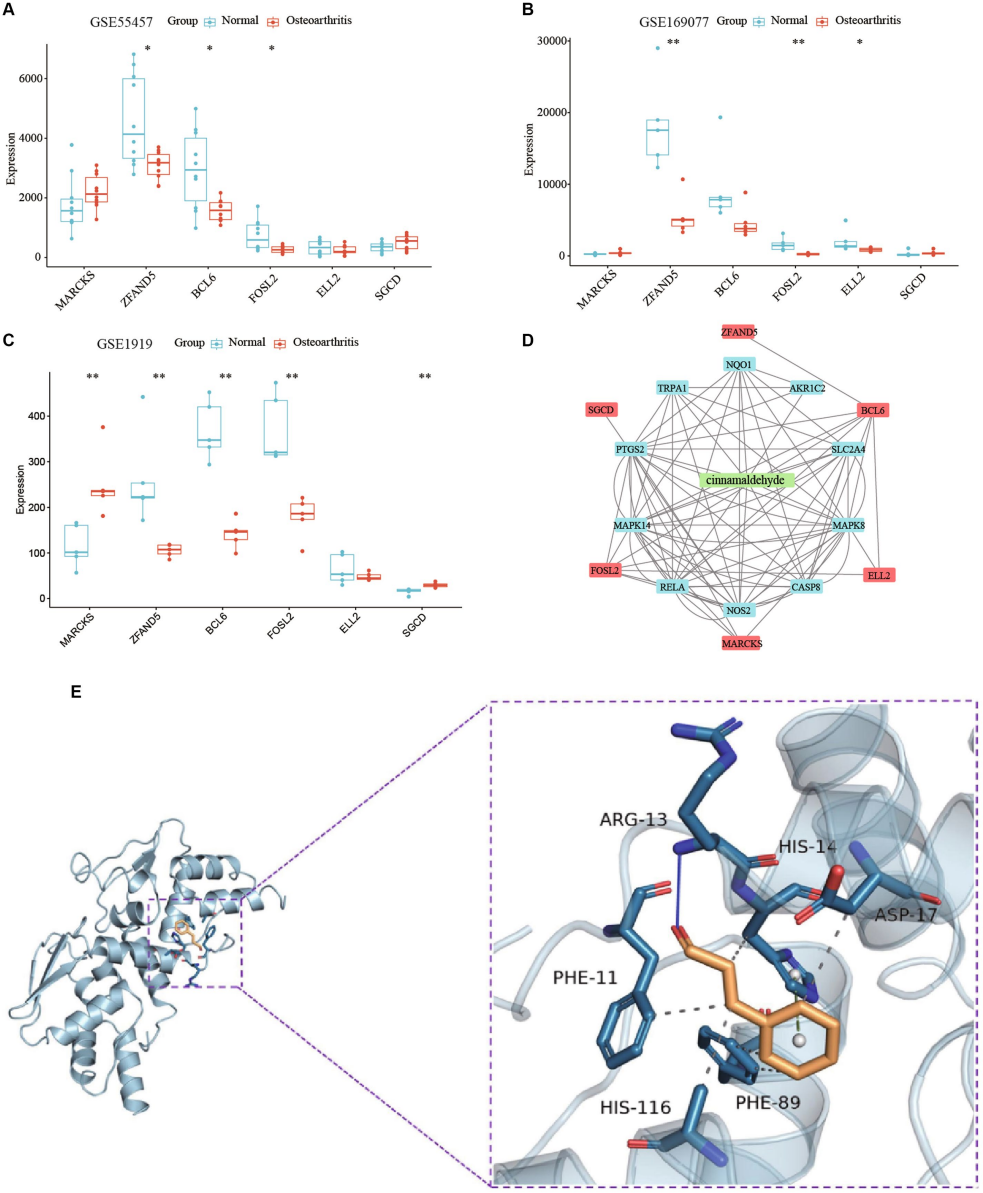
A study on the mechanism of cinnamaldehyde’s drug effectiveness. **(A)** The boxplot shows the expression of six key OA-related genes in GSE55457. **(B)** The boxplot shows the expression of six key OA-related genes in GSE169077. **(C)** The boxplot shows the expression of six key OA-related genes in GSE1919. **(D)** A network study on the effectiveness of cinnamaldehyde to OA. The six key OA genes are depicted in red; cinnamaldehyde is depicted in green; the cinnamaldehyde-interacting genes are depicted in blue. **(E)** Macromolecular docking diagram of active ingredient and key targets.

To validate correlations and predict the mechanism of the drug, we analyzed expression profiles from datasets GSE55457, GSE169077, and GSE1919. Expression analysis of *MARCKS*, *ZFAND5*, *BCL6*, *FOSL2*, *ELL2*, and *SGCD* between normal individuals and OA patients displayed trends consistent with our model and were further validated using an external dataset (Figures 10A–C). Integrated analysis using STITCH and STRING databases revealed interactions between cinnamaldehyde and 10 target genes *in vivo* (Figure 10D).

Molecular docking simulations assessed the binding affinity between cinnamaldehyde and the six target proteins, measured by binding energy. A binding energy below 0 kJ/mol suggests spontaneous binding, with energies less than −5.0 kJ/mol indicating high binding activity, and those below −7.0 kJ/mol indicating very strong binding activity. Cinnamaldehyde displayed a binding energy of −5.4 kcal/mol kJ/mol with BCL6, denoting a high affinity (Figure 10E). Interactions primarily occurred via hydrogen bonding and hydrophobic forces, with specific bonds and stacking detailed. Then, SPR experiments results verified that cinnamaldehyde and BCL6 belonged to the “fast binding/fast dissociation” kinetic characteristics (Supplementary Figure S4). The equilibrium dissociation constant (KD) of cinnamaldehyde and BCL6 was estimated at 9.09 × 10^−5^ M (Supplementary Figure S4), indicating that cinnamaldehyde binds to BCL6 directly.

Thus, cinnamaldehyde may target BCL6 directly and modulate other target genes indirectly, demonstrating its potential to regulate these key proteins in OA.

## 4 Discussion

Osteoarthritis (OA) is a debilitating joint disease, increasingly prevalent due to an aging population. No pharmacological treatments currently reverse the progression of OA, and management strategies primarily focus on alleviating symptoms. Emerging research suggests that targeting the primary drivers of OA progression, such as chondrocyte apoptosis, inflammation, and oxidative stress, through pharmacological interventions could improve treatment outcomes (30). Targeting chondrocyte apoptosis has been highlighted as a promising strategy to regulate cartilage degeneration (31). Furthermore, our Western blotting (Wb) (Supplementary Figure S3) and Immunofluorescence (Supplementary Figure S2) findings corroborate this by demonstrating consistency between the protein expression levels of the six target proteins and their RNA expression in IL-1β-stimulated chondrocytes. Notably, bioactive molecules like cinnamaldehyde, with their anti-apoptotic effects and safety profiles, are emerging as potential interventions for OA. Our study supports the potential for cinnamaldehyde to serve as an alternative or supplement to traditional medications by regulating chondrocyte apoptosis, offering a more comprehensive and sustainable approach to OA treatment.

The primary aim of this research was to explore whether cinnamaldehyde could mitigate senescence and apoptosis in osteoarthritic chondrocytes and to elucidate the mechanisms through which cinnamaldehyde may treat knee osteoarthritis (KOA). Our findings indicate that cinnamaldehyde treatment promoted the expression of the anti-apoptotic gene *Bcl-2* and suppressed pro-apoptotic gene *Bax* expression. The TUNEL and ROS assays revealed a significant reduction in apoptotic cells and cellular ROS expression levels in OA chondrocytes following treatment with cinnamaldehyde, suggesting its potential to inhibit chondrocyte apoptosis.

Additionally, we analyzed differentially expressed genes between normal and OA chondrocytes, identifying six significant apoptosis-associated genes: *ZFAND5*, *BCL6*, *ELL2*, *FOSL2*, *MARCKS*, and *SGCD*. Integrating results from prior studies (GSE55235 and GSE114007), we identified 55 differentially expressed genes associated with apoptosis. Our WGCNA analysis related specific gene modules to OA, and LASSO analysis pinpointed *MARCKS*, *ZFAND5*, *BCL6*, *FOSL2*, *ELL2*, and *SGCD* as significant model genes with robust confirmatory ROC curves from three independent datasets (GSE114007, GSE55457, GSE12021). PCR results from OA cells and patient plasma indicated a regulatory effect of cinnamaldehyde on these genes, notably reversing observed expression trends and interacting with cellular pathways involved in apoptosis.

Treatment with cinnamaldehyde reversed these trends. *SGCD*, a gene highly expressed in skeletal and cardiac muscles associated with dilated cardiomyopathy, is involved in the Ca^2+^ signaling, which directly influences apoptosis induction in tumor cells (32). In our results, *SGCD* showed down-regulation in OA chondrocytes and the blood of OA patients; however, its expression level exhibited a dose-dependent increase after cinnamaldehyde treatment. This finding contradicted results from bioinformatics analysis, possibly due to the influence of NSAIDs administered to patients in the datasets or sequencing bias.

Interestingly, prior studies have not reported the association of *ZFAND5*, *ELL2*, and *SGCD* with OA chondrocyte apoptosis, whereas other research identified *FOSL2* (33), *MARCKS* (34), and *BCL6* (35) as critical in OA progression. Additionally, cinnamaldehyde was shown to reverse gene expression levels in OA chondrocytes, supporting its potential therapeutic efficacy.

We hypothesize that *ZFAND5* may influence apoptosis through its well-documented ability to regulate the protein SLC3A2. As a member of the zinc finger AN1-type domain family, *ZFAND5* is predominantly expressed in heart and brain tissues (36) and modulates the expression of *SLC3A2* (37), a key protein in amino acid transport across cell membranes. Previous studies have established that *SLC3A2* inhibits vascular smooth muscle cell (VSMC) proliferation and promotes apoptosis (38). Therefore, we posit that diminished *ZFAND5* expression could upregulate *SLC3A2*, consequently augmenting total/lipid reactive oxygen species (ROS) levels, thereby promoting of chondrocyte apoptosis.

The role of *ELL2* in apoptosis regulation, while not definitively established, can be hypothesized from existing literature. ELL2 contributes to DNA damage regulation, participating in the KU protein complex and recognizing sites of double-strand breaks (DSBs) in DNA (39). DNA damage repair is crucial for preventing genomic mutations that might lead to cell cycle arrest, apoptosis, and OA pathogenesis (40). Therefore, a reduction in *ELL2* protein levels might result in unrepaired DNA damage, subsequently leading to chondrocyte apoptosis (41).

The identification of *SGCD* as critical to apoptosis can be attributed to its role in mitigating mitochondrial swelling and regulating the expression of the pro-apoptotic protein Bax. Research in *SGCD*-knockout mice demonstrated increased mitochondrial swelling and elevated Bax protein levels compared to wild-type mice, signifying the activation of the Bax-mediated mitochondrial-dependent cell death pathway (42). Inhibiting Bax slowed the progression of muscle symptoms in these mice, highlighting the pivotal influence of Bax and Bak proteins on cellular necrosis and apoptosis (43).

*MARCKS*, a substrate of protein kinase C, is implicated in cytoskeletal movement processes and has been linked to apoptosis in prostate cancer cells (44, 45). Notably, *MARCKS* also inhibits the expression of p38, JNK MAPKs, and NF-κB (46, 47).

FOSL2 has been demonstrated to regulate the formation of inflammasomes, directly associating with osteoarthritis chondrocytes (48).

Mechanistically, *BCL6* recruits *SIRT1* to the TP53 promoter, modulating histone acetylation and transcriptionally repressing TP53 expression. Early research indicated that miRNA-10a-5p targeting *BCL6* gene impacts proliferation, differentiation, and apoptosis in chicken myoblasts, with TP53 reduction attenuating apoptosis in OA cells.

When considering the genes studied and osteoarthritis treatment, it is essential to elucidate their clinical significance. Firstly, the identified APDEGs—*MARCKS*, *ZFAND5*, *BCL6*, *FOSL2*, *ELL2*, and *SGCD*—may provide deeper insights into osteoarthritis pathogenesis. Alterations in the expression or mutations of these APDEGs may reveal mechanisms underlying the onset and progression of osteoarthritis. Secondly, these APDEGs hold potential as diagnostic biomarkers that, by assessing mutation statuses or expression levels, could enable more precise, timely diagnosis and therapeutic interventions.

In clinical practice, APDEGs serve to enhance the accuracy of risk assessments for disease progression. Genetic testing could identify patients with specific gene variants, predicting the severity and progression rate of osteoarthritis and enabling more targeted treatment approaches. For example, patients identified at high risk may benefit from early physiotherapy and preventative pharmacotherapy. Moreover, APDEGs could be instrumental in stratifying treatment modalities where genetic variability among patients can lead to differential responses to therapies. Leveraging genetic information might thus enable the development of individualized and precision-based treatment plans to optimize therapeutic outcomes.

Looking forward, APDEGs are poised to become novel therapeutic targets. Investigating their functions and regulatory mechanisms could yield significant advances in osteoarthritis treatment, including the development of APDEG-targeted therapeutics, such as cinnamaldehyde derivatives. Additionally, the exploration of APDEGs could foster multidisciplinary collaboration, integrating expertise from genetics, molecular biology, clinical medicine, and related fields to innovate osteoarthritis management. Gene therapy also holds promise as a future intervention, wherein gene vectors could introduce functional genes into patients’ cells, potentially repairing or replacing defective genes and addressing genetic defects underlying osteoarthritis at their root.

## 5 Conclusion

In conclusion, this study presents novel evidence that cinnamaldehyde can effectively inhibit apoptosis in knee osteoarthritic chondrocytes, identifying specific gene targets through comprehensive bioinformatics analysis. The previously undocumented relationship between OA and the genes *ZFAND5*, *ELL2*, and *SGCD* underscores their potential as targets for future OA therapies, pointing toward new avenues for research and treatment modalities aimed at mitigating this prevalent and challenging disease.

## Data Availability

Publicly available datasets were analyzed in this study. This data can be found here: https://www.ncbi.nlm.nih.gov/geo/query/acc.cgi?acc=GSE55235; https://www.ncbi.nlm.nih.gov/geo/query/acc.cgi?acc=GSE114007; https://www.ncbi.nlm.nih.gov/geo/query/acc.cgi?acc=GSE55457; https://www.ncbi.nlm.nih.gov/geo/query/acc.cgi?acc=GSE12021; https://www.ncbi.nlm.nih.gov/geo/query/acc.cgi?acc=GSE169077; https://www.ncbi.nlm.nih.gov/geo/query/acc.cgi?acc=GSE1919.

## Footnotes

^1^ https://www.ncbi.nlm.nih.gov/geo/query/acc.cgi?acc=GSE55235

^2^ https://www.ncbi.nlm.nih.gov/geo/query/acc.cgi?acc=GSE114007

^3^ https://www.ncbi.nlm.nih.gov/geo/query/acc.cgi?acc=GSE55457

^4^ https://www.ncbi.nlm.nih.gov/geo/query/acc.cgi?acc=GSE12021

^5^ https://www.ncbi.nlm.nih.gov/geo/query/acc.cgi?acc=GSE169077

^6^ https://www.ncbi.nlm.nih.gov/geo/query/acc.cgi?acc=GSE1919

^7^ https://pubchem.ncbi.nlm.nih.gov/

^8^ http://www.rcsb.org/pdb/home/home.do

^9^ http://autodock.scripps.edu/

## Supplementary material

SUPPLEMENTARY FIGURE S1 **(A)** Analysis of apoptosis in chondrocytes using flow cytometry. **(B)** Protein level of apoptosis-related protein (BCL-2). β-actin was used as a control.

SUPPLEMENTARY FIGURE S2 Representative immunofluorescence images showing expression of ZFAND5, ELL2, BCL6, MARCKs, FOSL2, and SGCD (Scale bar = 100 μm).

SUPPLEMENTARY FIGURE S3 Protein level of different proteins (ZFAND5, ELL2, BCL6, MARCKs, FOSL2, SGCD, and BCL-2). β-actin was used as a control.

SUPPLEMENTARY FIGURE S4 The SPR binding curves of different concentrations of cinnamaldehyde and BCL6.
