# Supplementary figures and images for: Enhanced understanding of cinnamaldehyde’s therapeutic potential in osteoarthritis through bioinformatics and mechanistic validation of its anti-apoptotic effect
Source: Front Med (Lausanne). 2024 Sep 23;11:1448937. doi: 10.3389/fmed.2024.1448937 (PMC11456544; doi:10.3389/fmed.2024.1448937)

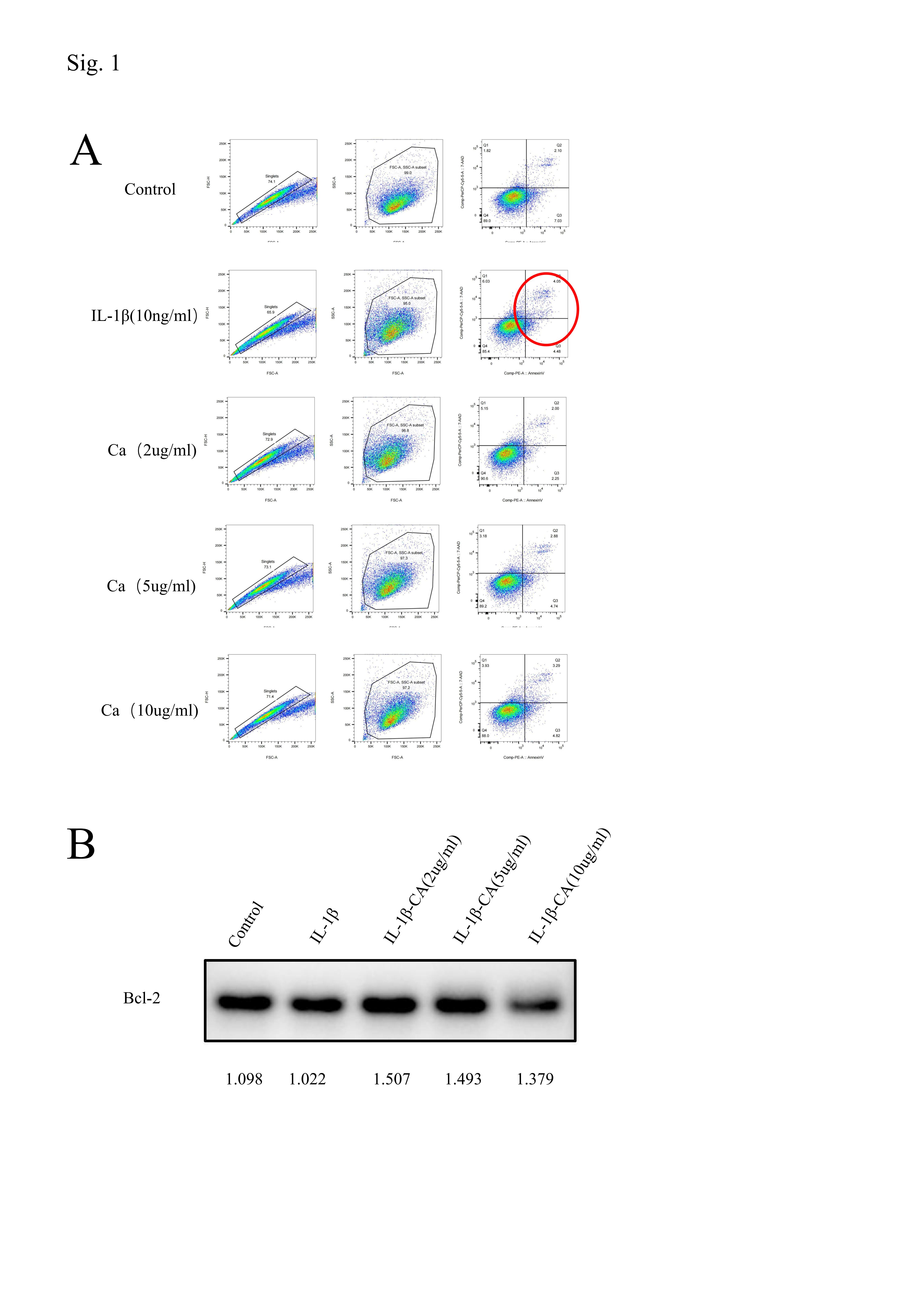

Supplement: SUPPLEMENTARY FIGURE S1 — (A) Analysis of apoptosis in chondrocytes using flow cytometry. (B) Protein level of apoptosis-related protein (BCL-2). β-actin was used as a control. [file Image_1.jpg]

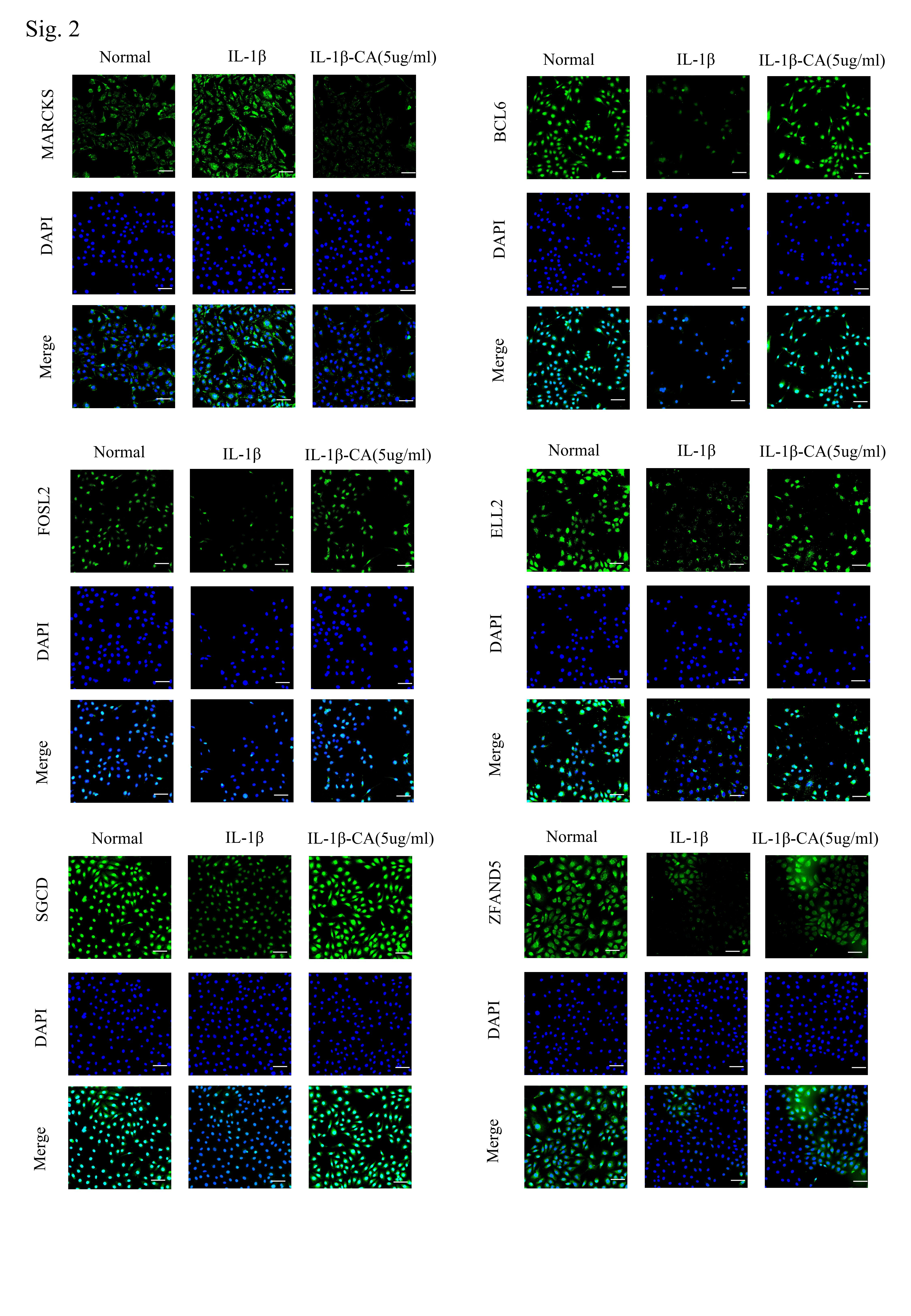

Supplement: SUPPLEMENTARY FIGURE S2 — Representative immunofluorescence images showing expression of ZFAND5, ELL2, BCL6, MARCKs, FOSL2, and SGCD (Scale bar = 100 μm). [file Image_2.jpeg]

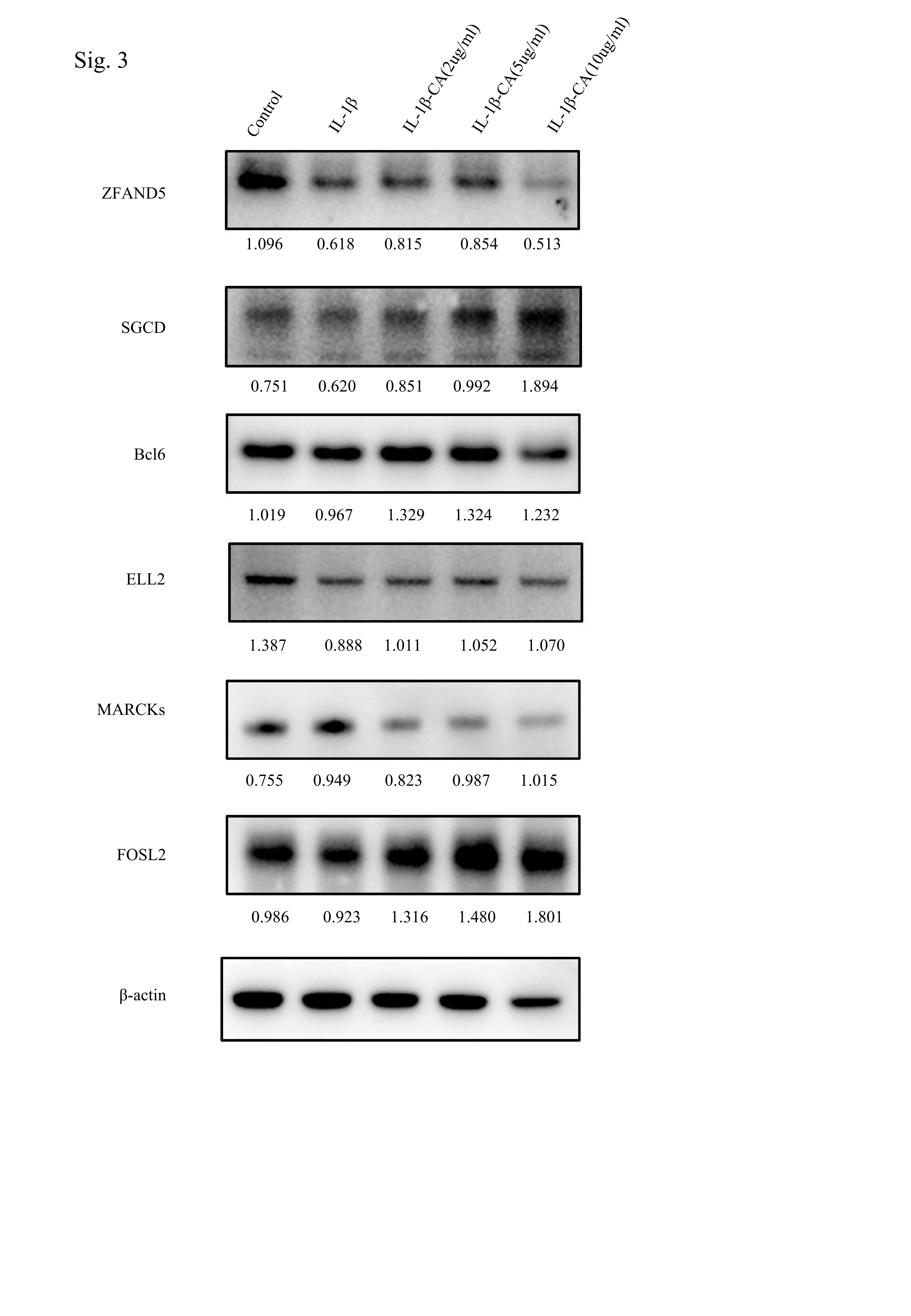

Supplement: SUPPLEMENTARY FIGURE S3 — Protein level of different proteins (ZFAND5, ELL2, BCL6, MARCKs, FOSL2, SGCD, and BCL-2). β-actin was used as a control. [file Image_3.jpeg]

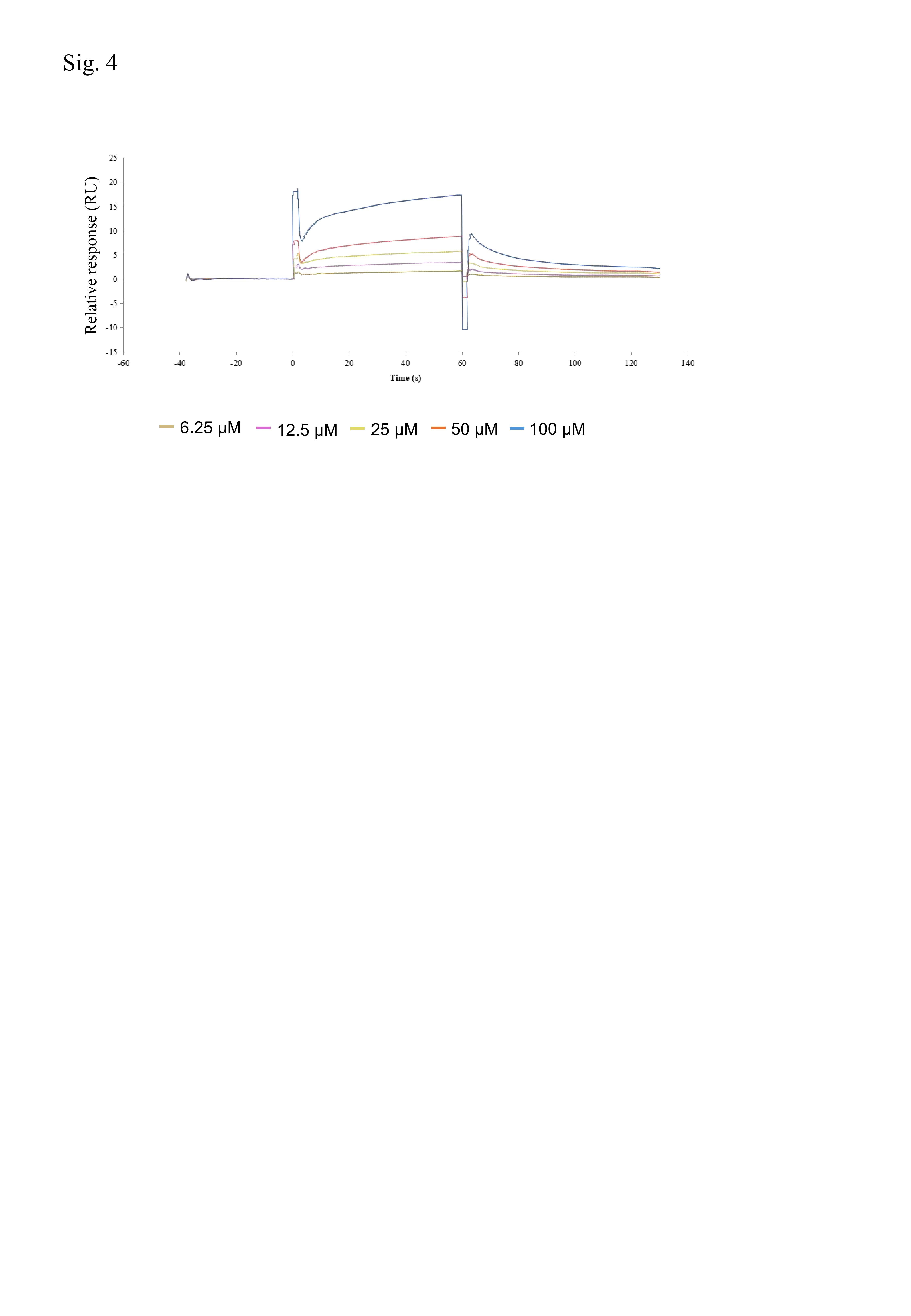

Supplement: SUPPLEMENTARY FIGURE S4 — The SPR binding curves of different concentrations of cinnamaldehyde and BCL6. [file Image_4.jpeg]
